# Silicosis: geographic changes in research: an analysis employing density-equalizing mapping

**DOI:** 10.1186/1745-6673-9-2

**Published:** 2014-01-17

**Authors:** Alexander Gerber, Doris Klingelhoefer, David A Groneberg, Matthias Bundschuh

**Affiliations:** 1 Institute of Occupational, Social and Environmental Medicine, Goethe-University, Theodor-Stern-Kai 7, Haus 9b, Frankfurt am Main 60590, Germany

**Keywords:** Silicosis, Scientometria, Density-equalizing mapping, H-Index, Citationrate

## Abstract

**Background:**

A critical evaluation of scientific efforts is needed in times of modified evaluation criteria for academic personnel and institutions.

**Methods:**

Using scientometric benchmark procedures and density-equalizing mapping, we analysed the global scientific efforts on “silicosis” of the last 92 years focusing on geographical changes within the last 30 years, specifying the most productive authors, institutions, countries and the most successful cooperations.

**Results:**

The USA as the most productive supplier have established their position as center of international cooperation, followed in considerable distance by the United Kingdom, Germany and China. Asian countries, particularly China, catch up and are expected to excel the USA still in this decade.

**Conclusion:**

The combination of scientometric procedures with density-equalizing mapping reveals a distinct global pattern of research productivity and citation activity. Modified h-index, citationrate and impact factor have to be discussed critically due to distortion by bias of self-citation, language and co-authorship.

## Introduction

Silicosis is a fibrotic lung disease caused by inhalation of crystalline silica (Silicon dioxide, SiO2). Universalized insufficient technical protection measures in the past to this day, result in exposition of countless workers over considerable spans of their lives and have made silicosis one of the most important occupational diseases worldwide [1]. The highly fibrogenic crystalline silica is the most abundant mineral worldwide. Of the several crystalline forms quartz, tridymite and cristobalite are the most important ones. Quartz occurs naturally in rocks such as granite, slate and sandstone, as well as in desert sand, which is practically pure silica. Tridymite and cristobalite are even more fibrogenic than quartz and occur naturally as high temperature polymorphs of silica, for example in lava [2]. For occupational purpose, silica is commonly used in the ceramic- and glass-industry, in foundries, chemical industry, electrical industry, natural stone industry, jewellery industry and for working methods such as sand jet polishing, abrasive technics with sand and many more [3]. Although protective measures have resulted in a declining in death rate due to silicosis in developed countries over the past decades, new outbreaks still occur in emerging nations [4,5]. Upon inhalation of respirable silica dusts, the crystals are deposited in the distal airways, and reactive oxygen species are produced on both the particle surfaces and via phagocytic cells. Phagocytosis of crystalline silica causes damage of the phagolysosomal membranes and induces apoptosis. Activation of the NALP3-Inflammasome triggers the inflammatory cascade by production of inflammatory cytocines, subsequently leading to fibrosis [6-8]. Diagnosis of silicosis generally requires a substantial occupational or environmental exposure of respirable cristalline silica and compatible radiologic abnormalities. Other competing diagnoses like infections, carcinomas, idiopathic interstitial pneumonia or interstitial lung diseases associated with a rheumatic disease have to be excluded. Chronic silicosis, the most common form of this disease, is distinguished from the accelerated silicosis and the acute silicosis by its low severity and rapidity of progression and usually develops after 10 to 30 years of low concentrated exposure to silica dust [5,9,10]. Patients may initially be asymptomatic and show shortness of breath at later stages. Chest radiography usually shows small round opacities, especially in the upper lung zones, and occasionally diffuse interstitial pattern of fibrosis which may be progressive over years, even after cessation of exposition. With retraction of the lung tissue, compensatory emphysema arises. The hilar and mediastinal lymph nodes often enlarge and frequently calcify in an eggshell pattern. Pulmonary function testing may be without pathological findings or reveals airflow limitation, restrictive defects and reduced diffusion capacity [9]. Accelerated silicosis develops 5–10 years after initial exposure to higher concentrations of crystalline silica dust. Clinical symptoms and radiologic imaging are similar to chronic silicosis but tend to progress more rapidly [11]. Acute silicosis, also known as silicoproteinosis, develops after exposure to high concentrations of respirable crystalline silica dust for a fiew weeks to 5 years. Most affected by acute silicosis are sandblasters and quartzite millers [5,12]. Radiographicaly a ground-glass appearance and air-bronchogramm is described. Dry cough, fever, fatigue, weight loss, respiratory insufficiency, and even death within months occur. Silicosis has been associated with several disorders, of which tuberculosis, COPD and lung cancer are the most important [13,14]. A curative treatment for silicosis does not exist, treatment options focus on preventing complications. In recent years, enormous efforts have been made to improve the understanding of pathogenetic mechanisms in silicosis. Particularly the finding of the NALP3-Inflammasome as major trigger of the inflammatory cascade appears to be an important step to break down into immunologic aspects of the disease and could explain the frequently observed incidence of rheumatic diseases e.g. rheumatoid arthritis or systemic lupus erythematodes as a consequence of breaking the immunologic tolerance [15-18]. Nevertheless, a scientometric approach to the topic “silicosis” has not been available despite an increased need to evaluate quality and quantity of scientific accomplishments. The aim of the present study is to evaluate the scientific effort in silicosis, considering geographical aspects, using large scale data analysis, scientometric approaches and density-equalizing procedures.

## Methods

### Scientometrics

Scientometrics is the science of measuring and analyzing research. It uses computational approaches and bibliometric methods such as citation analysis, impact factor or h-index.

### Data source

Data was retrieved from the database Web of Science (WoS) by Thomson Reuters and from the Medline database (PubMed) by the U.S. National Library of Medicine.

### Search strategy

Using PubMed and the MeSH search tags, MeSH heading related to “silicosis” were created, combined with the Boolean operator OR and entered as follows into the WoS search field to approximate the overall number of published items: “Silicosis” OR "Anthracoses" OR "Coal Worker's Pneumoconiosis" OR "Coal Worker Pneumoconiosis" OR "Coal Worker's Pneumoconioses" OR "Coal Workers Pneumoconiosis" OR "Pneumoconioses, Coal Worker's" OR "Pneumoconiosis, Coal Worker's" OR "Pneumoconiosis, Coal Worker" OR "Pneumoconiosis, Coal Workers" OR "Black Lung" OR "Black Lungs" OR "Lung, Black" OR "Lungs, Black" OR "Coal Miner's Lung" OR "Coal Miner Lung" OR "Coal Miner's Lungs" OR "Coal Miners Lung" OR "Miner's Lung, Coal" OR "Miner's Lungs, Coal" OR "Black Lung Disease" OR "Black Lung Diseases" OR "Coalworker's Pneumoconiosis" OR "Coalworker Pneumoconiosis" OR "Coalworker's Pneumoconioses" OR "Coalworkers Pneumoconiosis" OR "Pneumoconioses, Coalworker's" OR "Pneumoconiosis, Coalworker's". Further investigations used the “Analyse Results” function and the “Citation Report” provide by the WoS database.

#### Timeframe

The analysed timeframe covers the years from 1920 to 2012. Results from 2013 were not considered due to incomplete data acquisition at the time of analysis.

### Data analysis and categorization

Citation analysis is used as a tool to estimate quantitative and qualitative research productivity for the scientific community. All publications in the space of time referring to silicosis were analysed with the “citation report” method. Total number of citations and the average citation per item (citation rate) were calculated. Publication date, country of origin, source title, author and institution were other aspects of analysis. Findings were transferred to excel charts and illustrated in diagrams. Density-equalizing mapping procedures based on Gastner`s and Newman`s algorithm were used to visualize the distribution of the total number of published items and the average citation rate in a country-specific manner [19]. Using this approach, territories (countries) were resized in proportion to selected variables (for our purpose the number of published items and average citation rate). For methodical reasons, we decided to attribute any special administrative or autonomous region worldwide to its related country based on the present governmental status in the year 2013. E.g. Hong Kong was grouped to Mainland China despite its being a British colony until its reversion to China in 1997, and its medical system as well as the publications still being independent from Mainland China. Northern Ireland, Wales and Scotland were grouped to the UK and the former “German Democratic Republic” and the “Federal Republic of Germany” were grouped to Germany. This expectedly caused some distortion in each case for the benefit of the incorporating country and must therefore be regarded as a methodological bias.

#### H-index

The h-index is a bibliometric device, developed by Hirsch in 2005, to measure productivity and impact of scientists, institutions or countries at a point of time. It is calculated from the set of most cited publications and the number of received citations. The height of the h-index suggests the influence of the author in his scientific field [20,21]. For this study we used a modified h-index, generated on the basis of the authors work in the analyzed timeframe referring to silicosis. Only publications listed in the WoS in peer-reviewed journals with an impact factor were considered. Book articles were not considered as well as publications, when referring not explicitly to silicosis.

#### Impact factor

The impact factor was devised by Eugene Garfield, the founder of the Institute for Scientific Information, to estimate a journals relative importance in its scientific field. It reflects the average number of citations per annum, published articles received during the two preceding years [22].

#### Analysis of cooperation

To investigate cooperation between countries and institutions, data of references were gained from WoS as plain text files and analysed. If at least two authors, coming from different institutions or countries, contributed to a publication, this relationship was defined as a cooperation. To visualize the productivity of cooperation for each pair of institutions or countries, a vector was calculated, proportional to the number of cooperation in thickness and shade of grey.

## Results

### Total number of published items, analysis of origin and cooperation

**Figure 1:**
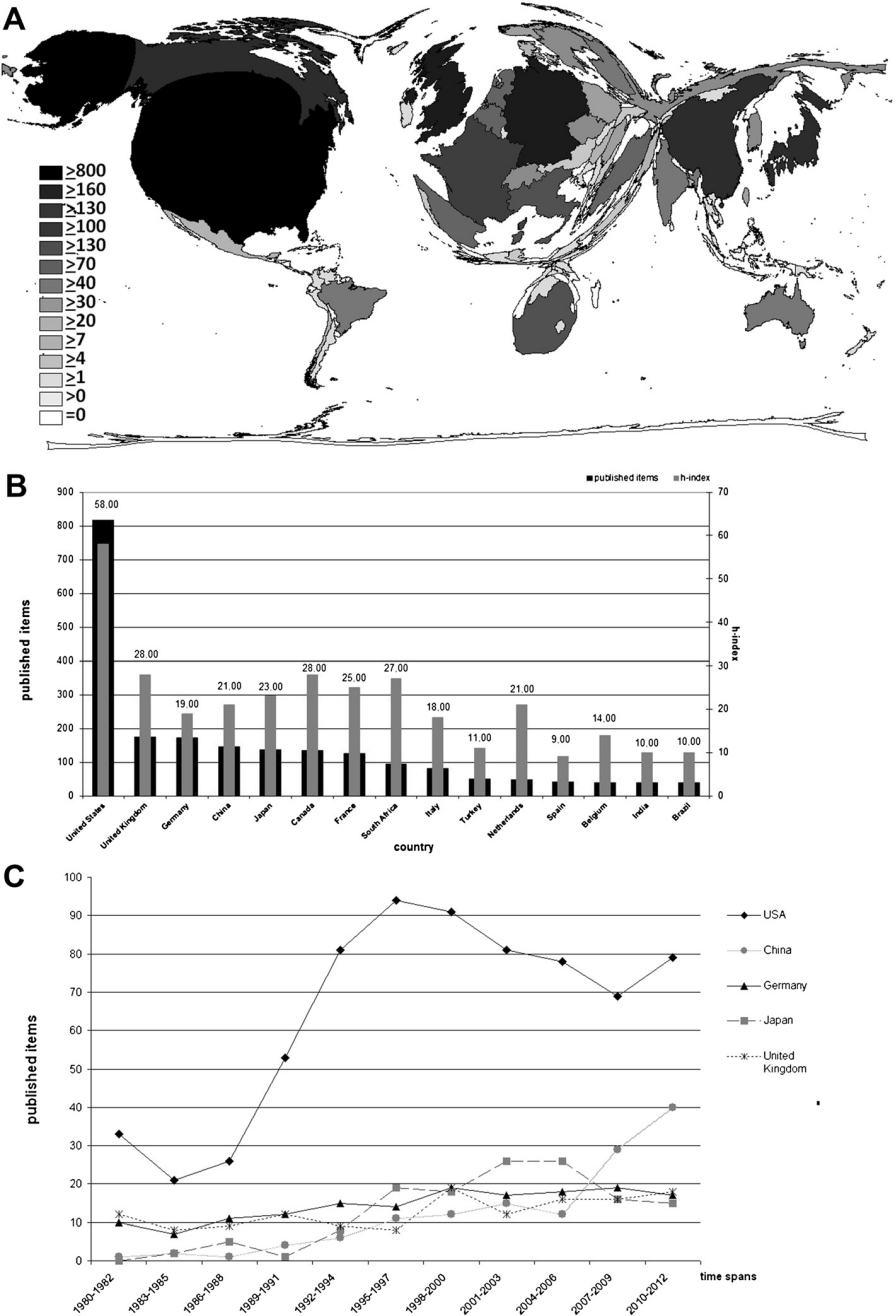
**Published items regarding "silicosis". A:** Density-equalizing map illustrating the number of publications in each particular country (the area of each country and its colour were scaled in proportion to its total number of publications regarding "silicosis" as illustrated in the panel). **B:** Ranking of country total number of published items an h-index related to "silicosis". **C:** Trend of the scientific productivity of the five most productive countries.

**Figure 2:**
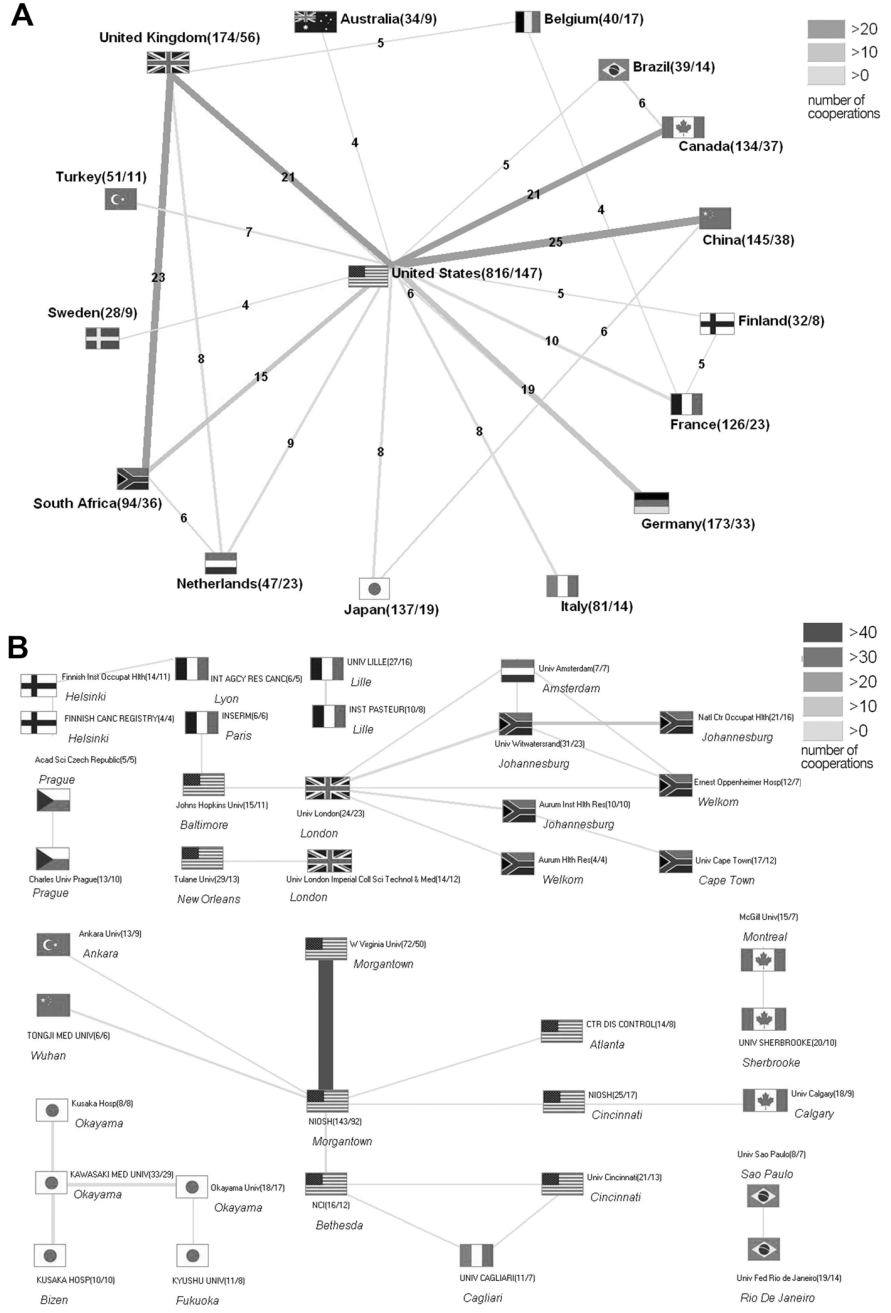
**Cooperating articles. A:** Analysis of the international cooperations. **B:** Analysis of the interinstitutional cooperations.

The WoS database provided a total of 2,805 published items with a reference to silicosis in the research timeframe. The publications originate from 74 countries with the USA being the most productive supplier (Figure 1A). Overall, US-American scientists participated in 29% of all publications, followed by researchers from the United Kingdom, Germany, China and Japan respectively (Figure 1A + B). Only the USA published more than 500 items hence their dominating the cartogram. Major parts of South America (except for Brazil), Africa (except for South Africa) and East-Asia (except for Japan and China) are minimized. An analysis of developments in the country-specific scientific productivity since 1980 detects remarkable changes in geographic foci. While China started contributing measurably to the worldwide scientific output related to silicosis as late as only in 1980, it has since developed into the currently second most productive country with a tendency to rise (Figure 1C). Most of the scientific efforts are concentrated in the USA. According to this, the USA were placed in the middle of the diagram (Figure 2A). The cooperations between the USA and China, the USA and the United Kingdom, the United Kingdom and South Africa, the USA and Canada, and between the USA and Germany have been identified as the most productive ones. The closest inter-institutional collaboration worldwide exists between the West Virginia University Hospital in Morgantown (USA) and the National Institute for Occupational Safety and Health (NIOSH) in Morgantown (USA) (Figure 2B).

### Citation parameters

**Figure 3:**
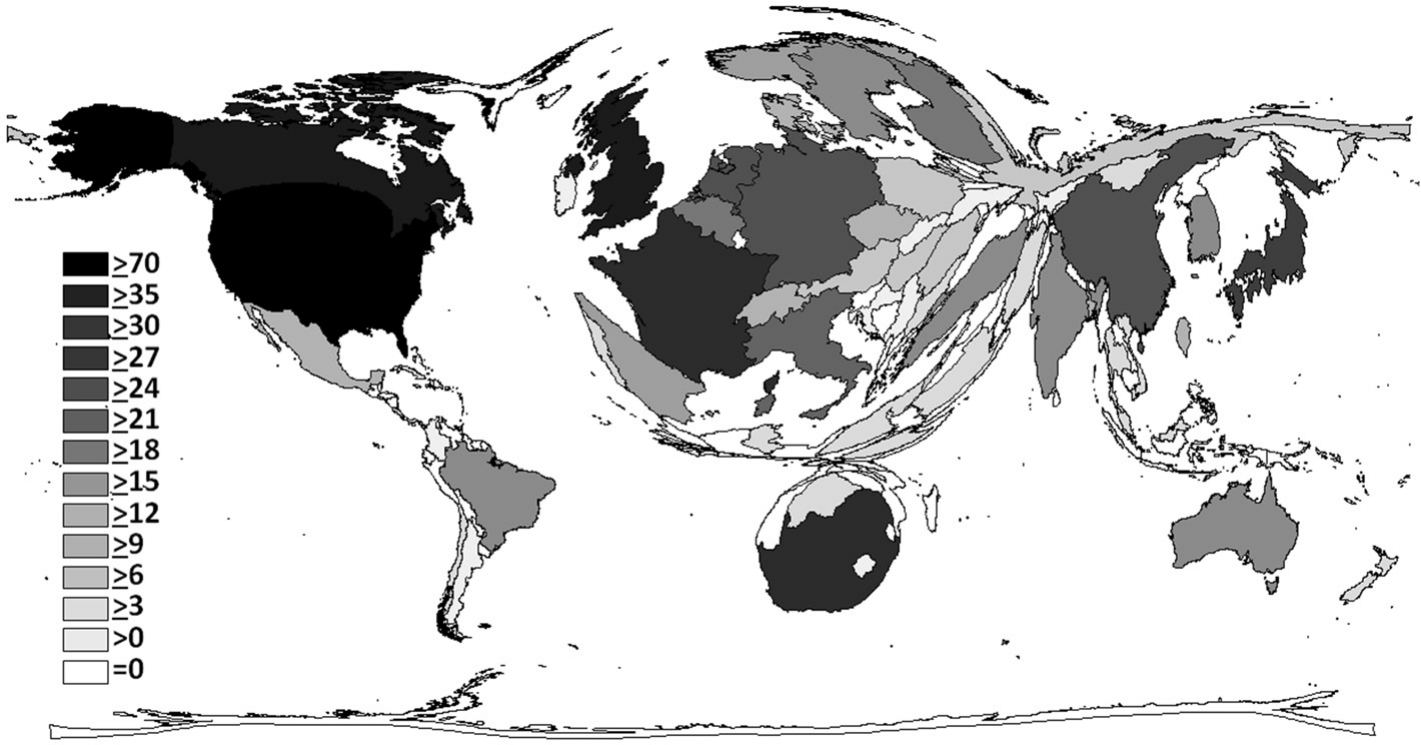
**Density-equalizing map showing the h-index of each particular country.** The area of each country was scaled in proportion to its h-index regarding “silicosis”.

**Figure 4:**
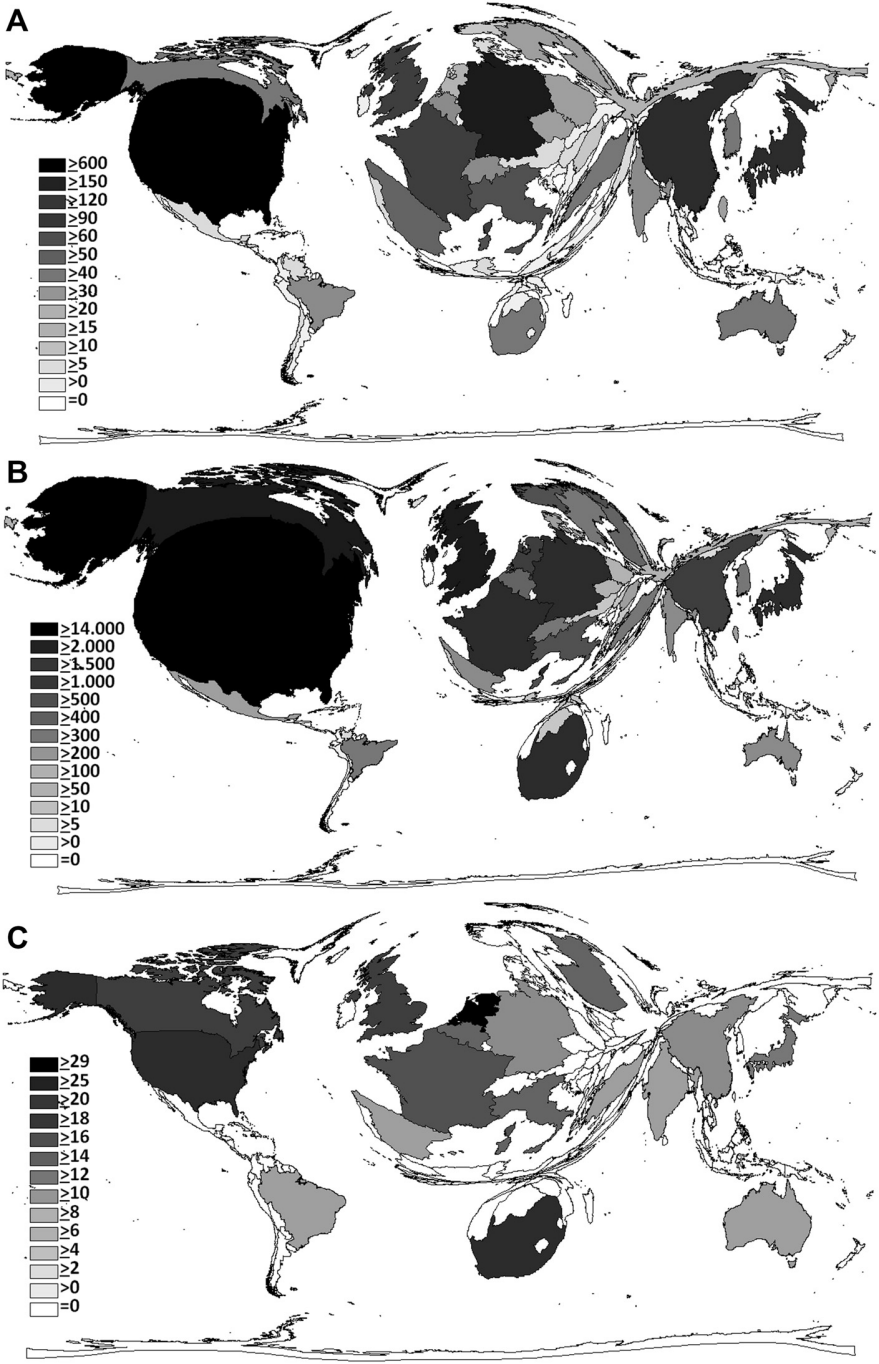
**Number of scientific institutions engaged with "silicosis" (A), number of citations received concerning "silicosis" (B) and citations rate (C) of each particular country. A:** Density-equalizing map showing the number of scientific institutionsconcerned with the topic “silicosis” of each particular country. The area of each country was scaled in proportion to its number of institutions. **B:** Density-equalizing map illustrating the total number of citations each country received concerning “silicosis”. The area of each country was scaled in proportion to its number of received citations. **C:** Density-equalizing map illustrating the citation rate (average citation per publication) of each particular country. The area of each country was scaled in proportion to its citation rate. Threshold > 30 published items.

The United States of America (58), hold the highest h-index in a country-specific manner, followed by the United Kingdom and Canada (both 28), and South Africa (27) (Figure 1B + Figure 3). The highest number of research institutions engaged with the topic “silicosis” is agglomerated in the USA, followed by Germany, Canada, China and Japan (Figure 4A). The USA also receive the highest total amount of citations, followed with considerable distance by Canada and the UK (Figure 4B). As for the citation rate (CR, average citation per publication), the Netherlands attract attention (Figure 4C) with a CR of 23 (47 published items, 1,092 received citations) outnumbering even the most productive and frequently cited countries such as South Africa (CR: 19), the USA (CR:18), Canada (CR: 17) and the UK (CR:16).

### Analysis of publishing journals and the most productive authors concerned with silicosis

**Figure 5:**
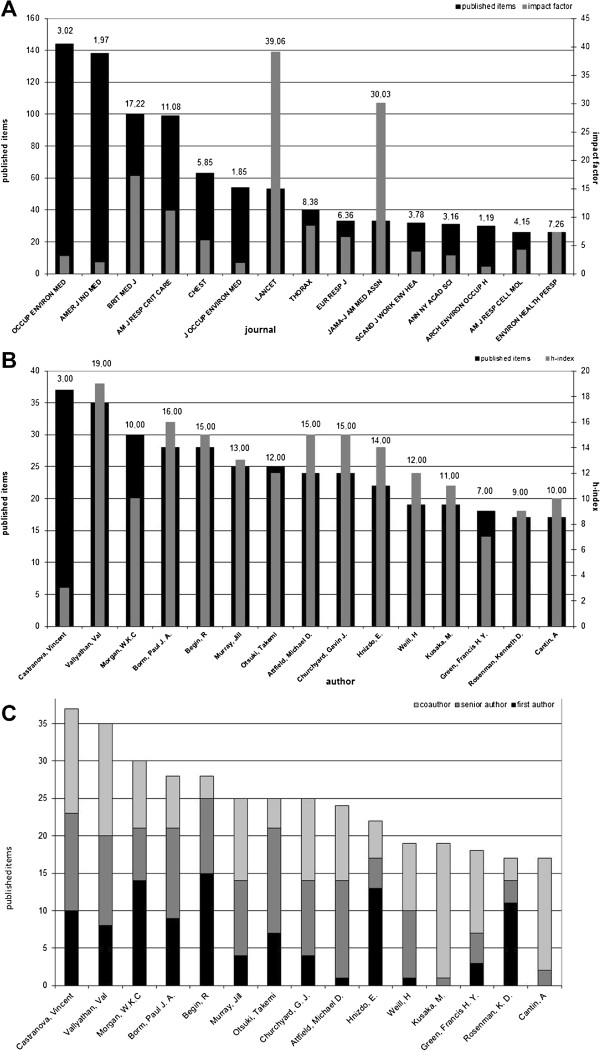
**Top 15 journals (A), most productive authors (B) and their proportion of first- authorship and senior-authorship to co-authorship. A:** Top 15 ranking of journals by the number of published items and their impact factor items during the period 1920–2012 **B:** 15 most productive authors and their h-index in the period 1920-2012 **C:** 15 most productive authors considering the proportion of first-authorships and senior-authorships to co-authorships.

Identified as the most productive journal dealing with silicosis (Figure 5A), “Occupational and Environmental Medicine”, established in 1944 as the “British Journal of Industrial Medicine” leads with 144 published items and an impact factor of 3.02, followed by the “American Journal of Industrial Medicine” with 138 items (impact factor 1.97) and the “British Medical Journal” (impact factor 17.22), publishing 100 items. Furthermore, the ten most productive authors and their h-index were determined (Figure 5B + C). “Castranova, Vincent” (NIOSH, Morgantown, USA) is the most productive author with 37 published items in the analysed time span, followed by “Vallyathan, V.” (NIOSH, Morgantown, USA) with 35 items, “Morgan, W.K.C” (Canada) with 30 items and Borm, Paul JA (Netherlands) with 28 items. The h-index shows a slightly different distribution, as “Vallyathan, V.” holds the highest h-index (19), followed by “Borm PJA” (16). Regarding first- and senior-authorship, “Begin, R” with 14 first- and 10 senior-authorships leads by far and shows the best ratio of first- and senior- authorships to co-authorships. To get valid results for all parameters, we checked and corrected each author, institution and country, mentioned in the WoS, for misspelling, variations in name spelling, affiliations and fusions, renaming or unification, usually by making inquiries via world wide web.

## Discussion

In the present study, bibliometric tools and density-equalizing procedures were used to evaluate quantitative and qualitative aspects of the scientific output in the field of “silicosis”. After a decrease of worldwide research productivity for about ten years beginning in the late 1990ies, new findings in recent years about the NALP 3- Inflammasome and immunologic aspects of silicosis seem to have caused an increase of scientific activity. The USA maintain their predominant position as illustrated in Figure 1A, but Asian countries, particularly China, catch up and are expected to excel the USA still in this decade. The presently outstanding productivity of the USA does not surprise as they dispose the highest agglomeration of research institutions and provide an exemplary scientific infrastructure (Figure 4A). As expected, the USA show the most intensive collaboration behaviour on a national as well as on an international level (Figure 2A + B), of which the most productive notably proved to be the one with China. As international projects tend to have more influence on the scientific community, an intensive collaboration behaviour on an international level increases the likelihood to produce research of higher international relevance, usually leading to higher numbers of received citations in comparison with a collaboration on a lower international or only national level [23,24]. This might explain the dominating position of the Netherlands even compared to the USA and the other most productive countries, when considering only the citation rate but not the h-index or the total number of citations which both are limited by the total amount of publications (Figure 4C). In comparison with their overall scientific output, the Netherlands cooperate intensively on an international level, predominantly with the USA, the UK and South Africa (Figure 2A). Also the forth most productive author worldwide with the second highest h-index is Dutch. To avoid overestimation of a few articles that may have been cited often, we used a threshold of 30 publications for the calculation of the citationrate. In times of growing competition in the scientific community, modified evaluation criteria for academic personnel and a rising need to publish, a subsequent tendency to co-authorship and author self-citation is seen which can artificially inflate an authors productivity and disproportionately affect impact factor and h-index. Several studies and simulations recently have proven that particularly studies with more authors published in lower profile journals are most vulnerable to this effect [25-27]. A recent correspondence in “The Lancet” discussed this problem with regards to the evaluation-practice of medical doctors in China [28,29]. However, after analysing the ratio of first- and senior-authorship to co-authorship of the most productive authors, as well as analysis of self-citations, using the WoS citation report for the most productive countries, biases of co- authorship or self-citation have not turned out to be the reason for the success of the ten most productive authors and countries. To estimate a journals relevance in its scientific field, the impact factor was used as a parameter for the scientific quality of a publishing journal. In this regard, the “British Medical Journal” and the “American Journal of Respiratory and Critical Care Medicine” have the best results (Figure 5A). Apparently, journals explicitly focusing on the field of occupational and environmental medicine as “Occupational and Environmental Medicine” and the “American Journal of Industrial Medicine” are inferior to clinical journals in publishing the most relevant items. It should be mentioned that the subsequent rise of the impact factor among English-language journals results in more citations leading to a language bias [30]. This is supported by the fact that all 15 most productive journals publishing on the subject “silicosis” are in English language. However, scientometric indicators such as h-index, citationrate and impact factor, commonly used for assessment of scientific quality, have to be seen critically due to distortion by self-citation, co-authorship and language-bias. To exclude these biases automatically from calculations of h-index, productivity and impact factor, a further scientometric approach is needed.

## Competing interests

The authors declare that they have no competing interests.

## Authors’ contributions

DAG and MB made substantial contributions to the conception and design of the study, acquisition of the data data and have been involved in drafting and revising the manuscript. DK contributed to creating the figures. All authors have read and approved the final manuscript.
